# The Effects of Different Exercise Programmes on Female Body Composition

**DOI:** 10.2478/hukin-2014-0091

**Published:** 2014-11-12

**Authors:** Rosa Maria Soares Costa de Mendonça, Adenilson Targino de Araújo Júnior, Maria do Socorro Cirilo de Sousa, Helder Miguel Fernandes

**Affiliations:** 1 University of Trás-os-Montes and Alto Douro, Vila Real, Portugal.; 2 Federal Institute of Technology Education, Campina Grande, Paraíba, Brazil.; 3 Laboratory of Kinanthropometry and Human Performance, Federal University of Paraíba, Brazil.; 4 Research Centre for Sport, Health and Human Development; Vila Real, Portugal.

**Keywords:** health, physical exercise, anthropometry, strength training, dance, hydrogymnastics

## Abstract

The purpose of this study was to verify the effects of 16 weeks of practicing different exercise programmes on body composition. This is an exploratory and descriptive study of 89 women aged 25 to 55 years (41.42 ± 9.23 years). The subjects were randomly divided into three experimental groups (EG): practitioners of strength training (SG), dance (DG), hydrogymnastics (HG), and a control group (CG) with sedentary women. Measurements of body mass and height, circumferences of the chest, waist, abdomen, hips, thighs, calves, and skinfolds of the triceps, suprailiac and thigh were registered in three different moments: prior to the commencement of the training program, again after 8 weeks of training, and finally after 16 weeks of training. Body density was estimated by using the trifold protocol by Jackson, Pollock and Ward. The ANOVA and deltas of change (Δ%) were used for data analysis. The level of significance was set at p<0.05. The effects of greater statistical significance on body composition related the variables “time”, “group” and the interaction between the two (time × group) were observed for the percentage of fat - F% (F (1.79, 152.52) = 24.59, p <0.001, η ^2^ = 0.22), fat mass - FM (F (1.75, 149.01) = 12.65, p <0.001, η ^2^ = 0.13) and lean mass - LM (F (1.77, 150.66) = 47.38, p <0.001, η ^2^ = 0.36). The HG and SG were more beneficial in reducing F%. It was observed that the EG indicated healthier anthropometric aspects compared to the CG, regardless of the type of exercise programmes practiced. The time factor was more representative over the effects of exercise on anthropometric dimensions.

## Introduction

Regular practice of physical exercise has positive effects on body composition and functional abilities, whether or not exercised is combined with dietary control. This has been proven by empirical and scientific literature, especially in female subjects (Colado et al., 2009; De Glisezinski et al., 2003; Fagherazzi et al., 2013; Hagger and Chatzisarantis, 2005; Hojan et al., 2013; Monteiro et al., 2004; Ross et al., 2004; Van Aggel-Leijssen et al., 2002).

Considering the characteristics of the study sample with regard to health, a woman does not have significant changes in body composition until the period of menopause (Fagherazzi et al., 2013; Hojan et al., 2013). The transition period to menopause is not consistently associated with weight gain, which is influenced more by the lack of exercise and the consumption of alcohol (Moreira and Sardinha, 2003). More active premenopausal women have lower amounts of subcutaneous fat, as well as a more favourable distribution (Bocalini et al., 2009; Bonganha et al., 2011; Bravo et al., 2013). This fact may be associated not only with a greater expenditure of energy, but also with other influential factors of energy balance, namely exercise and caloric consumption.

Previous data collection on overweight subjects in the Brazilian population was based on the body mass index (BMI). These data showed that the tendency towards weight gain (≥ 25 a ≤ 30 kg/m^2^) occurred in women around age of 25–34 (40.7%). This becomes more evident between the age of 55–64 (60.7%). Weight gain stabilises again after the age of 65 (59.1%), although the final weight is often higher than it was in early adulthood (Ministry of Health – Vigitel Brazil, 2013). Taking into consideration the aforementioned facts, there has been increased attention on lifestyle changes that can affect body composition. These changes include disease control and inducing desirable health changes (Colado and Triplett, 2008; Katula et al., 2006; Olinto et al., 2007; Rech et al., 2006). However, there has been little research into the clinical and functional responses of women who participate in combined physical activities (De Glisezinski et al., 2003; Ross et al., 2004; Dias et al., 2006; Van Aggel-Leijssen et al., 2002). Thus, there is a need for verification of the effects of different types of physical exercise compared with and related to anthropometric variables (Rejeski et al., 2001).

The use of only one type of activity for training is common. However, there are known natural limitations to any form of exercise that need to be addressed. In many cases, additional actions and movement adaptations are needed in order to compensate for the limitations and improve the individual’s performance. In this context, it is important that prescribed exercise programs effectively motivate and provoke positive changes in the components of body composition, such as reducing body fat and gaining lean mass, which are both important indicators of health.

There are still knowledge gaps that allow for the identification of changes in anthropometric dimensions, especially in body composition. Therefore, the following hypothesis of this study was derived:

It is possible to change body composition and anthropometric dimensions of adult women after 16 weeks of practicing different physical exercise programmes (strength training, dance or hydrogymnastics).

## Material and Methods

### Study Design

This study is a longitudinal, quasi-experimental study. The comparative analysis is done within groups and intra-groups and includes both pre and post-tests.

### Participants

The sample was comprised of 89 adult women aged 25–55 (41.42 ± 9.23 years). Of these, 60% were married, 27% single and 12% divorced, all residing in the northeastern part of Brazil. These women were selected using a non-probabilistic manner in specific locations, such as fitness clubs, hydrogymnastic gyms and a public municipal institution. The sample was randomly divided into four groups, of which one was designed as the control group consisting of individuals that were sedentary (CG) (n = 25) and three were characterised as experimental groups: strength training (SG) (n = 25), dance (DG) (n = 18), and hydrogymnastics (HG) (n = 21). The inclusion criteria were as follows: participating in the sessions of physical exercises arranged within this study, not suffering from infectious and chronic diseases nor musculoskeletal disorders, being a beginner or participant of physical activities for no more than 12 months prior to the study, and negative responses to the questions on the Readiness for Physical Activity Survey (RPA - S).

### Dependent variables: Anthropometric measures

In order to take measurements of the anthropometric variables, the subjects were informed about the requirement for performing a physical evaluation according to Sousa (2008). One of the study’s researchers took all of the measurements in the afternoon (14:00 to 16:00) in private rooms at the gym and in the public institution. Body mass was measured in kilograms (kg) using a Plenna scale model MEA-07420 with accuracy of 100 g and a range of 150 kg. Body height was measured in centimetres (cm) using a stadiometer from Personal Caprice Sanny ES2060 with precision of millimetres (mm) and height up to 2.20 metres. Chest circumference, as well as the circumference of the waist, abdomen, hip, thighs and calves were determined by using a measuring tape for T-87 Wiso body measurements. The skin fold – SF (mm) measurements of the triceps, suprailiac and thigh were taken using a fold compass (calliper Cescorf Scientific) with accuracy of 0.1 mm and a constant pressure of 10 g/mm^2^ at all openings.

Three measurements were taken for each variable, and then the average was used in order to perform the statistical analysis. The software *Physical Test 8.0,* with the Jackson, Pollock and Ward’s (1980) protocol 3DC for females aged 18 to 55 was used in order to equate the results of body composition through anthropometric measurements.

### Independent variables: Physical exercise protocols

#### Strength Training Group (SG)

The exercise prescription followed the ACSM recommendations (Garber et al., 2011), with a frequency of three times per week at moderate to vigorous intensity of 60 to 70% of 1RM. Three sets of 8–12 repetitions with a 2–3 min rest period and 50–60 min duration per session were performed. The major muscle groups of the upper and lower limbs were exercised with the use of either machines with weights, free weights or resistance equipment. An increase in the amount of weight used for each exercise was used when the participant was able to easily perform the activity. A physical education teacher guided the sessions, controlled the intensity and the volume, and corrected the posture of the participants during the exercises.

#### Dance Group (DG)

The exercise routines for the dance sessions followed the ACSM guidelines (Garber et al., 2011) with a frequency of three times per week at a moderate to vigorous intensity, which was defined as 60 to 85% of the maximum heart rate as identified by the calculation 220 – age, according to Karvonen’s proposal (Power and Howley, 2000). The sessions lasted from 50 to 60 minutes each. This type of activity involved the major muscle groups in a continuous manner using basic steps and a minimum of three rhythmic variations of popular dance styles and aerobics per session with songs of a rhythmic cadence of 100 to 160 beats per minute.

#### Hydrogymnastic Group (HG)

The hydrogymnastic practice was held in accordance to the ACSM guidelines (Garber et al., 2011) with a frequency of three days per week at moderate to vigorous intensity, defined as 60 to 85% of the maximum heart rate. The exercises involved the major muscle groups of the upper and lower limbs. The main component of this program included cardiorespiratory exercises, followed by muscular endurance exercises using equipment such as shin pads designed for hydrogymnastics, dumbbells, bars, plates, floating devices and pool edges. Each exercise took from 2 to 3 min to complete, with a total session time of 45 to 55 min.

### Procedures

This study complied with Resolution 466/12 of the National Health Council of 12/12/2012 (Brazil, 2012) regarding conducting research on human beings. In addition, the protocol was approved by the Liga Norte Riograndense Contra o Câncer Institutional Ethics Committee (embodied in document number 124/2011).

After approval was received, the sample recruitment was carried out in fitness clubs and hydrogymnastic gyms, as well as a public municipal institution for dance. The women were randomly divided into either one of three experimental groups (SG, DG and HG) or a control group (CG). All of the participants were invited to sign the Statement of Individual Consent Form (SICF). The study continued with meeting the women on the day and time scheduled in order to conduct the tests, which were carried out in the facilities of the participants’ own gyms.

The activities started at 14:00 with the measurement of the anthropometric variables. First, body mass was measured, followed by body height, then the circumferences and finally the skin folds. After completion of the first tests (pre-test), the women participated in a 16 week long intervention with exercises (strength training, dance, hydrogymnastics), with a reevaluation occurring after eight weeks (second test) and at the end of the study (after 16 weeks). This resulted in a total of three measurements taken at different times, using the same procedures.

The pre-test data were collected from 104 women. During the pretest period and the eight-week re-evaluation, 6 participants withdrew from the study. In addition, another 9 participants withdrew between the 8^th^ and 16^th^ weeks. Most of the participants of the control group that dropped out did not give a reason, nor did they attend the evaluation sessions. In the experimental group, the main reason given for withdrawing from the study was temporary absence from the exercise routine for personal reasons or complete abandonment of the exercise programmes. There was no sample loss due to illness or death. The third and final data collection after 16 weeks was conducted with 89 participants.

All of the physical activity routines followed by the experimental groups (EG) included regular visits from the researcher as well as regular communication with their teachers for each modality in the facilities in which they developed their own programmes.

### Statistical Analysis

The data were analysed using the SPSS version 20 programme in order to determine the descriptive statistics, including the average, standard deviation and inferential statistics of the dependent variables: body mass, waist circumferences, abdomen, hips, body mass index (BMI), fat percentage (%F), fat mass (FM), lean mass (LM) and waist-hip ratio (WHR). The effects of the variables time (beginning - 8 weeks - 16 weeks), group (strength training - dance -hydrogymnastics - sedentary) and interaction (time × group) were analysed for the anthropometric measurements using ANOVA for repeated measures. For each variable, the values of the magnitude of the differences (F value), the significance (p) and the estimated effect size (value of η^2^), with values greater than 0:01, 0:06, 0:14 respectively, representing small, medium and high effects (Pestana and Gageiro, 2005) are shown. In addition, the deltas of change (Δ%) were presented, and were calculated as follows: 100 * (T1 - T3) / T3, with a significance level of 5%.

## Results

No significant differences were observed for age, marital status, BMI, and menopause, as shown in Table 1. The only variables showing differences between the groups at baseline were the educational level (p < 0.05) and the time of physical practice (p < 0.01).

Given the differences in the education level between groups at baseline, the linear association between this variable and the assessed dependent anthropometric variables were analysed, yet no significant relationship (p> 0.05) was found. Thus, the variable of the education level was not included as a covariate.

For the group of anthropometric variables, only the WHR presented the assumption of data sphericity and the Greenhouse-Geisser correction was adopted for the remaining ones. Relative to body mass, there was an interaction effect (time × group) of (*F*_(4.27, 120.94)_ = 2.89, *p* = 0.023, η^2^ = 0.09), and an average effect for the time variable of (*F*_(1.42, 120.94)_ = 8.52, *p* = 0.001, η^2^ = 0.09). For the variable group (*F*_(3, 85)_ = 0.48, *p* = 0.698, η^2^ = 0.02), no effects on body mass were observed.

For waist circumference, an interactive effect of (*F*_(5.27, 149.17)_ = 2.26, *p* = 0.048, η^2^ = 0.07) occurred, as well as for the group variable, causing a significant effect of great size of (*F*_(3, 85)_ = 4.51, *p* = 0.006, η^2^ = 0.14). In other words, the different exercise programmes explain the variations in waist circumference. The group with the highest average in this variable was the one practicing hydrogymnastics. However, the sequence of measurements did not show large variations during the experiment because of the absence of effect on the time variable (*F*_(1.76, 149.17)_ = 0.83, *p* = 0.423, η^2^ = 0.01). Likewise, waist circumference also showed no significant effects, i.e. this variable did not change throughout the study.

The hip circumference showed a significant interaction of (*F*_(4.41, 125.07)_ = 3.75, *p* = 0.005, η^2^ = 0.12), despite the lack of interference of the exercise type (*F*_(3, 85)_ = 0.12, *p* = 0.951, η^2^ = 0.00). Small changes occurred between measurements, given the observed delta values (Table 2) and no effect of the time variable of (*F*_(1.47, 125.07)_ = 1.52, *p* = 0.225, η^2^ = 0.02) was found.

No interaction effects were found in the follow-up times waist-hip ratio (WHR). However, only the influence of the type of exercise was indicated, since the group effect was significant with a high effect size of (*F* (3, 85) = 7.83, *p* = 0.001, η^2^ = 0.22). The hydrogymnastic and sedentary groups had higher observed values (Table 2).

The effect of the interaction between groups and the body mass index (BMI) measurements (*F*_(5.91, 147.14)_ = 2.36, *p* = 0.041, η^2^ = 0.08) were observed as being of moderate size, as well as the intervention of time (*F*_(1.73, 147.14)_ = 6.67, *p* = 0.003, η^2^ = 0.07). However, an effect for the applied treatment with the exercises (*F*_(3, 85)_ = 1.13, *p* = 0.342, η^2^ = 0.04) was not observed.

The hydrogymnastics group showed the highest values for this measurement. However, the biggest changes were identified in sedentary women, with an increase of the variable during the research (Table 3).

The fat percentage (F%) showed an interaction effect (time × group) of (*F*_(5.38, 152.52)_ = 4.30, *p* =0.001, η^2^ = 0.13). The type of exercise performed during the 16 weeks had a high impact on the measurements (*F*_(3, 85)_ = 5.92, *_p_* = 0.001, η^2^ = 0.17). Only the sedentary group showed no decrease in these measurements (Table 3). The highest reductions were observed in the hydrogymnastics and strength training groups. In addition, the F% changed significantly over the three measurements (time effect) of (*F*_(1.79, 152.52)_ = 24.59, *p*<0.001, η^2^ = 0.22), with a high effect size and a decrease in relation to the initial values for all of the experimental groups.

There was an interactive effect of (*F*_(5.26, 149.01)_ = 3.93, *p* = 0.002, η^2^ = 0.12) for fat mass. Nevertheless, the observed changes in these measurements cannot be attributed to the interventions applied, given the absence of the group effect of (*F*_(3, 85)_ = 1.82, *p* = 0.149, η^2^ = 0.06). However, the sequence of measurements was significant with a moderate effect size of (*F*_(1.75, 149.01)_ = 12.65, *p* < 0.001, η^2^ = 0.13), and a decrease in fat mass for all groups except for the sedentary women was observed (Table 3).

No significant values were identified given (*F*_(5.32, 150.66)_ = 2.04, p = 0.071, η^2^ = 0.07) when evaluating the interaction of the two effects (time × group) for the measurements of lean mass. The practiced exercise form exerted a moderate effect on this measurement (*F*_(3, 85)_ = 3.03, p = 0.034, η^2^ = 0.10). To conclude, an increased effect of time (*F*_(1.77, 150.66)_ = 47.38, p < 0.001, η^2^ = 0.36) is observed for this variable, presenting an increase throughout the research, especially for the group that performed hydrogymnastic activities (Table 3).

## Discussion

The present study aimed to investigate whether or not different types of exercise programmes had an effect on women’s body composition. The investigation was made by using different experimental groups (strength training, dance or hydrogymnastics) and a control group (sedentary), based on the equation of anthropometric measurements that were recorded over the course of the supervised training programmes. In general, the comparative analysis results indicated that after 16 weeks of training, the women practicing physical activities (SG, DG and HG) revealed a healthier anthropometric profile, with lower levels of body fat, when compared to the sedentary group (CG) over time. Bravo et al. (2013) also reported similar results. They claimed that more active premenopausal women had lower values of subcutaneous fat and a more favourable fat distribution. This fact may be associated not only with higher energy expenditure, but also with other influential factors of energy balance, namely exercise and calorie consumption.

Among the analysed anthropometric variables, the following stand out as having a statistically significant effect: waist circumference, WHR, F%, FM and LM in the relationships between time, group and the interaction of time versus group. The group with the highest values of waist circumference, F% and WHR was the one practicing hydrogymnastics, revealing the closest anthropometric profile to the sedentary group. However, the measurements did not change significantly during the experiment, observing the absence of the time effect at the end of the 16 weeks. Mendonça et al. (2011) found in a previous study using a cross-sectional sample of 66 women that those practicing hydrogymnastics had a higher level of BMI, F%, WHR and waist circumference when compared to other exercising groups. Likewise, we considered that a possible answer may be that the condition that this sample revealed a higher average age (45.19 ± 8.81 years) than the other study participants, although, other factors may also contribute to explain this phenomenon.

Other peculiarities were found in a cross-sectional study with 981 Brazilian women, aged 20–60 years living in southern Brazil. The study investigated the effect of socioeconomic and demographic data, as well as the lifestyle in the occurrence of obesity, which was evaluated through the waist circumference. The highest prevalence of abdominal obesity was found in women over 50 with low education levels and increasing age (Olinto et al., 2007; Sarno and Monteiro, 2007). All of these findings are consistent with the already-mentioned studies, as well as those corroborated by the results of this research.

The literature reveals a dynamic body composition profile during aging (Colado et al., 2009; Moreira and Sardinha, 2003). Naturally, the women’s body composition profile changes become visible over the years, being most evident when approaching the climacteric period. During this period, weight gain, increased body fat, and changes in the composition and distribution of adipose tissues are observed. Hence, we considered the identification of the anthropometric profile, regarding the deposition of abdominal fat of adult, premenopausal, physically active, and sedentary women as important. In addition, we found that using waist circumference is an easy and low-cost measure in the use of population studies (Olinto et al., 2006). Such a variable may be compared with other anthropometric indicators as being the best predictor of visceral fat located in the abdominal region (Rankinen et al., 2006), showing a strong correlation with the majority of the metabolic risk factors (Fox et al., 2007; Olinto et al., 2007).

Particular body composition fraction effects of higher statistical significance were observed in body fat percentage (F%) and fat mass (FM). It showed similar effects over time, hence, representing changes of this component in the three stages of analysis in the period of 16 weeks. The type of exercise also helped in changing the body composition of physically active women, taking into account that the control group of sedentary individuals was the only group that showed no reduction in these measurements. The largest reductions were observed in the hydrogymnastic and strength training groups. Although, there may be other factors to explain these results, we believe that a possible explanation might be related to the characteristics of each modality, the intensity of exercise and the increase and control of weights, compared to the group that practiced dance. The observed changes in fat mass cannot be attributed to the interventions applied, given the absence of a significant group effect.

This finding is enhanced by Stuck et al.’s (2012) study analysing the effects of supervised strength training and physical abilities programmes on the body composition of 77 postmenopausal women. The subjects were divided into a control group and three additional groups using three different types of devices: machines with weights, elastic devices and aquatic devices that increase the drag force. One of the most surprising results was the great reduction of body fat when using weight machines as compared to elastic bands. As the authors observed, the group exercising with elastic bands reduced their body fat by 1.93% and the group using the weight machines reduced theirs by 5.15%. On the other hand, the group using aquatic devices showed a body fat reduction of 2.5%. The authors reported that programmes that use weight machines can be more effective in reducing fat mass in a short-term; however, more studies are needed to confirm this hypothesis, as there were small differences in the relationship of this variable between the groups at the time of the pretest. Colado and Triplett (2008), however, did not observe these differences.

Likewise, the lean mass component demonstrated an elevated effect of the time of intervention, showing that, in general, the practice provided positive changes, independent of exercise mode. This favoured an increase in lean body mass over the 16 weeks, especially for the hydrogymnastics group. In the hydrogymnastics group, there was a moderate effect of the variable in the intervention group for this measurement. In the variable of interaction, however (time × group), no significant values were identified.

Velthuis et al. (2009) allowed us, through their study, to establish some similarities with the results of the present investigation. They conducted a survey of 189 sedentary postmenopausal women, aged 50–59 years, in order to verify the effect of 12-month programmes with aerobic and strength training components on body composition. In that study, 96 women were assigned to an exercise practicing group and 93 to the control group. The evaluations were carried out in three stages (initial, four months and 12 months), and the parameters measured were as follows: body mass, body height (body mass index), waist-hip circumference, body fat distribution and lean mass. According to the results, the exercise programmes resulted in no significant effects on the body mass index and the hip circumference. The exercise group achieved a greater loss in total body fat compared with the control group. In addition, lean body mass increased significantly (0.31 kg), while the hip circumference (0.57 cm) decreased, as compared to the control group. The study concluded that the exercise programmes that combined aerobic and strength exercises for 12 months in postmenopausal women did not affect body mass, yet, they still influenced the distribution of body fat, as well as the waist circumference. This measure can have important implications for health as a risk factor for obesity.

In addition, we would like to mention some limitations of this research. First, there was no control of daily habits in any of the groups. In the future, this could be addressed through a questionnaire. Likewise, we did not control dietary intake, which is a confounding variable in this type of research. Regarding the anthropometric measurements, there was a lack of use of gold-standard equipment in order to collect the data, as well as an absence of reliability measurements for the tests. Despite these failures, the proposed exercise programmes had an impact on the analysed components, according to our results.

Based on the results of the present study, we can conclude that the time factor was the most representative of the effects on the anthropometric dimensions of adult women practicing strength training, dance or hydrogymnastics after 16 weeks. This was specifically evident in the measurements of waist circumference, waist-hip ratio, body fat percentage, fat mass and lean mass. Physically active women possess a healthier anthropometric profile when compared to sedentary ones, regardless of the type of exercise performed. The practitioners of strength training and hydrogymnastics had a greater reduction of body fat percentage as compared to those practicing dance. Therefore, studies with a longer observation period than 16 weeks can estimate effects of higher power, not only in the variable of the duration of the intervention, but also in the characteristics of the group variables, as well as the interaction between time and group. This in turn enables a more comprehensive view of the benefits of the exercises on the anthropometric dimensions in women.

## Figures and Tables

**Table 1:** Characteristics of participants at baseline (N=89)

**Groups**	**Strength training (n=25)**	**Dance (n=18)**	**Hydrogymnastics (n=21)**	**Sedentary (n=25)**	***F/X*^2^**
**Age (years)**	38.84 ± 9.97	41.50 ± 8.70	45.19 ± 8.81	40.76 ± 8.62	1.92

**Scholarly Levels (%)**					

5th to 9th grade Fundamental Incomplete	12.0	0.0	0.0	0.0	
Fundamental Complete	8.0	0.0	14.3	8.0	21.05*
Medium Complete	52.0	16.7	38.1	28.0	
University Graduate	28.0	83.3	47.6	64.0	

**Marital Status (%)**					

Single	36.0	22.2	23.8	24.0	
Married	56.0	61.1	71.4	56.0	4.46
Divorced	8.0	16.7	4.8	20.0	

**Time Physical Activity (months)**	7.60 ± 4.66	4.00 ± 3.63	5.86 ± 5.02	0.00 ± 0.00	17.83**

**Body Mass Index (kg/m^2^)**	25.50 ± 5.02	24.88 ± 3.95	27.25 ± 5.22	25.27 ± 3.96	1.08

**Symptoms of Menopause**					

Yes	24.0	33.3	57.1	36.0	5.58
No	76.0	66.7	42.9	64.0	

**Socioeconomic Status (Points in CCEB, 2012)**	25.40 ± 6.50	30.00 ± 5.52	27.86 ± 6.34	25.44 ± 6.72	2.50

Note:

* p<0.05,

** p<0.01

**Table 2:** 

**Variables**	**Time 1 (T1) Beginning**	**Time 2 (T3) 8 weeks**	**Time 3 (T3) 16 weeks**	**Δ1% (T2–T1)**	**Δ2% (T3–T2)**
	M±DP	M±DP	M±DP		
**Body mass (kg)**					
Strength training (n=25)	65.79 ± 11.33	66.18 ± 10.90	65.58 ± 10.44	0.59	−0.91
Dance (n=18)	63.38 ± 9.78	63.97 ± 9.64	64.19 ± 9.61	0.93	0.34
Hydrogymnastics (n=21)	67.71 ± 12.00	67.98 ± 11.74	68.41 ± 12.06	0.40	0.63
Sedentary (n=25)	64.39 ± 11.93	65.34 ± 11.91	66.18 ± 11.95	1.48	1.29

**Waist Circumference (cm)**					
Strength training	76.30 ± 7.17	75.95 ± 6.51	75.95 ± 6.40	−0.46	0.00
Dance	76.96 ± 7.91	77.19 ± 7.45	76.92 ± 7.71	0.30	−0.35
Hydrogymnastics	85.37 ± 10.50	84.19 ± 10.74	84.37 ± 11.30	−1.38	0.21
Sedentary	80.16 ± 8.79	80.36 ± 8.71	81.02 ± 8.98	0.25	0.82

**Abdominal Circumference (cm)**					
Strength training	86.41 ± 7.96	86.25 ± 7.83	85.73 ± 7.47	−0.19	−0.60
Dance	87.39 ± 8.19	86.83 ± 8.50	87.03 ±7.67	−0.64	0.23
Hydrogymnastics	93.40 ± 10.46	92.16 ± 10.53	91.70 ± 10.31	−1.33	−0.50
Sedentary	90.24 ± 9.14	90.60 ± 8.32	90.88 ± 8.90	0.40	0.31

**Hip Circumference (cm)**					
Strength training	100.76 ± 8.42	99.92 ± 7.98	99.28 ± 7.38	−0.83	−0.64
Dance	99.29 ± 6.83	99.01 ± 6.75	99.03 ± 6.93	−0.28	0.02
Hydrogymnastics	100.74 ± 8.27	101.08 ± 8.93	99.66 ± 9.12	0.34	−1.40
Sedentary	99.75 ± 6.98	99.88 ± 7.11	100.85 ± 7.28	0.13	0.97

**Waist-Hip Ratio**					
Strength training	0.76 ± 0.04	0.76 ± 0.04	0.77 ± 0.03	0.00	1.32
Dance	0.78 ± 0.05	0.78 ± 0.05	0.78 ± 0.05	0.00	0.00
Hydrogymnastics	0.85 ± 0.08	0.83 ± 0.07	0.84 ± 0.08	−2.35	1.20
Sedentary	0.80 ± 0.07	0.81 ± 0.07	0.80 ±0.07	1.25	−1.23

Descriptive analysis of anthropometric variables

**Table 3:** Continuation of descriptive analysis of anthropometric variables

**Variables**	**Time 1 (T1) Beginning**	**Time 2 (T3) 8 weeks**	**Time 3 (T3) 16 weeks**	**Δ1% (T2–T1)**	**Δ2% (T3–T2)**
	M±DP	M±DP	M±DP		
**Body Mass Index BMI (kg/m^2^)**					
Strength training (n=25)	25.50 ± 5.02	25.45 ± 4.67	25.31 ± 4.42	−0.20	−0.55
Dance (n=18)	24.88 ± 3.95	25.17 ± 3.90	25.35 ± 3.90	1.17	0.72
Hydrogymnastics (n=21)	27.24 ± 5.22	27.42 ± 5.13	27.63 ± 5.30	0.66	0.77
Sedentary (n=25)	25.27 ± 3.96	25.69 ± 3.91	26.01 ± 3.99	1.66	1.25

**Fat Percentage (%)**					
Strength training	25.72 ± 7.07	23.95 ± 6.26	23.63 ± 5.76	−6.88	−1.34
Dance	29.92 ± 4.34	28.98 ± 4.20	28.31 ± 4.35	−3.14	−2.31
Hydrogymnastics	31.76 ± 5.56	30.57 ± 5.38	28.99 ± 5.44	−3.75	−5.17
Sedentary	29.47 ± 5.72	29.30 ± 5.75	29.56 ± 4.55	−0.58	0.89

**Fat Mass (kg)**					
Strength training	17.54 ± 7.93	16.37 ± 7.08	15.97 ± 6.51	−6.67	−2.44
Dance	19.35 ± 5.85	18.84 ± 5.48	18.47 ± 5.48	−2.64	−1.96
Hydrogymnastics	21.99 ± 7.94	21.20 ± 7.18	20.28 ± 7.32	−3.59	−4.34
Sedentary	19.59 ± 6.89	19.66 ± 7.12	20.01 ± 6.48	0.36	1.78

**Lean Mass (kg)**					
Strength training	48.24 ± 4.76	49.80 ± 5.09	49.60 ± 4.95	3.23	−0.40
Dance	44.08 ± 4.72	45.12 ± 4.87	45.71 ± 5.04	2.36	1.31
Hydrogymnastics	45.67 ± 4.92	46.76 ± 5.69	48.09 ± 5.84	2.39	2.84
Sedentary	44.79 ± 5.72	45.67 ± 5.64	46.15 ± 5.76	1.96	1.05
